# Stability of SARS-CoV-2 RNA in Nonsupplemented Saliva

**DOI:** 10.3201/eid2704.204199

**Published:** 2021-04

**Authors:** Isabel M. Ott, Madison S. Strine, Anne E. Watkins, Maikel Boot, Chaney C. Kalinich, Christina A. Harden, Chantal B.F. Vogels, Arnau Casanovas-Massana, Adam J. Moore, M. Catherine Muenker, Maura Nakahata, Maria Tokuyama, Allison Nelson, John Fournier, Santos Bermejo, Melissa Campbell, Rupak Datta, Charles S. Dela Cruz, Shelli F. Farhadian, Albert I. Ko, Akiko Iwasaki, Nathan D. Grubaugh, Craig B. Wilen, Anne L. Wyllie, the Yale IMPACT Research team

**Affiliations:** Yale School of Public Health, New Haven, Connecticut, USA (I.M. Ott, A.E. Watkins, C.C. Kalinich, C.A. Harden, C.B.F. Vogels, A. Casanovas-Massana, A.J. Moore, M.C. Muenker, M. Nakahata, A.I. Ko, N.D. Grubaugh, A.L. Wyllie);; Yale School of Medicine, New Haven (M.S. Strine, M. Boot, M. Tokuyama, A. Nelson, J. Fournier, S. Bermejo, M. Campbell, R. Datta, C.S. Dela Cruz, S.F. Farhadian, A.I. Ko, A. Iwasaki, C. Wilen);; Howard Hughes Medical Institute, New Haven (A. Iwasaki)

**Keywords:** 2019 novel coronavirus disease, coronavirus disease, COVID-19, severe acute respiratory syndrome coronavirus 2, SARS-CoV-2, viruses, respiratory infections, zoonoses, diagnostics, saliva

## Abstract

The expense of saliva collection devices designed to stabilize severe acute respiratory syndrome coronavirus 2 RNA is prohibitive to mass testing. However, virus RNA in nonsupplemented saliva is stable for extended periods and at elevated temperatures. Simple plastic tubes for saliva collection will make large-scale testing and continued surveillance easier.

Despite increased diagnostic testing capacity for severe acute respiratory syndrome coronavirus 2 (SARS-CoV-2), testing in many countries, including the United States, is still inadequate for slowing the coronavirus disease (COVID-19) pandemic. Many persons still do not have access to SARS-CoV-2 testing, and for some that do, an imbalance between supply and demand at large testing centers leads to long delays before results are received. The demand for testing will only increase as many schools, colleges, and workplaces reopen. Ideally, specialized population surveillance–oriented testing would require minimal diversion of resources from clinical diagnostic testing, be affordable and scalable, and enable rapid and reliable virus identification for persons with asymptomatic or subclinical infections. Thus, simplifying the sample collection and testing workflow is critical.

A simple solution is saliva collection. Saliva is a sensitive source for SARS-CoV-2 detection (*1*–*3*) and an alternative sample type for antigen and antibody testing (*4*,*5*). In addition, saliva collection is noninvasive, can be reliably performed without trained health professionals, and does not rely on a sometimes-limited swab supply. However, almost all saliva-based tests approved by the US Food and Drug Administration require specialized collection tubes containing stabilization or inactivation buffers that are costly and not always available. Moreover, as saliva continues to gain popularity as a potential specimen to aid testing demands, standardized collection methods have not been defined for saliva collection as they have for swab-based specimen collection. When true saliva is not collected (e.g., if it contains sputum), which can happen with COVID-19 inpatients when saliva is difficult to produce, specimens can be difficult to pipette (*6*). Combined with untested concerns regarding SARS-CoV-2 RNA stability in saliva, using supplements to reduce degradation and improve sample processing has become common. Previous work with saliva samples, however, has indicated that some buffers optimized for host nucleic acid stabilization may actually inhibit viral RNA detection (*7*) (S.B. Griesemer et al., unpub. data, https://doi.org/10.1101/2020.06.16.20133041), particularly in extraction-free PCRs (D.R.E. D.R.E. Ranoa et al., unpub. data, https://doi.org/10.1101/2020.06.18.159434). Thus, if true saliva (relatively easy to pipette) is being tested, the utility of collecting saliva in expensive tubes containing purported stabilization buffers comes into question. To explore the viability of broadly deploying affordable saliva-based surveillance approaches (*8*), we characterized SARS-CoV-2 RNA stability and virus infectivity in saliva samples stored in widely available, sterile, nuclease-free laboratory plastic (polypropylene) tubes.

## The Study

**Figure 1:**
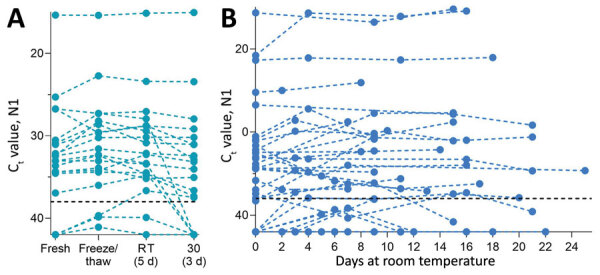
Stability of severe acute respiratory syndrome coronavirus 2 (SARS-CoV-2) RNA (N1) detection in saliva. A) Detection of SARS-CoV-2 RNA in 20 saliva samples on day of sample collection (fresh) did not significantly change after storage at −80°C (to assess the effect of a freeze/thaw cycle), 3 days at 30°C, or 5 days at RT (recorded as ≈19°C). Detection of N1 remained similar to that of freshly collected samples, regardless of starting C_t_ value (Pearson r = −0.085, p = 0.518). B) At RT, detection remained stable for up to 25 days. Colored dashed lines track the same sample through different storage conditions. Black horizontal dashed lines represent C_t_ 38, which we applied as the cutoff to determine sample positivity. Samples that remained not detected after 45 cycles are depicted on the x-axis. C_t_, cycle threshold; RT, room temperature.

We used saliva collected from COVID-19 inpatients and at-risk healthcare workers into sterile wide-mouth containers (*3*) without preservatives (nonsupplemented) to evaluate the temporal stability of SARS-CoV-2 RNA at different holding temperatures (−80°C, 4°C, ≈19°C, 30°C) (Appendix). SARS-CoV-2 RNA from saliva was consistently detected at similar levels regardless of the holding time and temperatures tested. After RNA extraction and quantitative reverse transcription PCR (qRT-PCR) testing for SARS-CoV-2 on the day of saliva collection (*3*), we aliquoted and stored the remaining 20 sample volumes at −80°C, room temperature (≈19°C), and 30°C. Whether stored at −80°C, room temperature (5 days), or 30°C (3 days), the qRT-PCR cycle threshold (C_t_) values for the N1 region of the nucleocapsid protein did not differ significantly from those for samples tested on the day of collection (Figure 1, panel A). After the freeze/thaw cycle or storage at room temperature, we observed C_t_ decreases of 1.058 (95% CI 2.289 to 0.141) for freeze/thaw and 0.960 (95% CI −2.219 to 0.266) for room temperature; however, the strength of this effect was low. We saw a similar effect after incubation at 30°C, with a C_t_ increase of 0.973 (95% CI −0.252 to 2.197). Moreover, SARS-CoV-2 RNA remained relatively stable in saliva samples left at room temperature for up to 25 days (C_t_ 0.027, 95% CI −0.019 to 0.071 C_t_) (Figure 1, panel B). Regardless of starting C_t_ value (viral load), this prolonged stability of SARS-CoV-2 RNA was also observed when samples were stored for longer periods at −80°C (maximum 92 days), 4°C (maximum 21 days), and 30°C (maximum 16 days) (Appendix Figure 1).

Although SARS-CoV-2 RNA from saliva remained stable over time, we observed a decrease in human ribonuclease P at higher temperatures (room temperature, C_t_ 1.837, 95% CI 0.468 to 3.188 C_t_; 30°C, C_t_ 3.526, 95% CI 1.750 to 5.349 C_t_; Appendix Figure 2); the change in concentration was greater than that observed for SARS-CoV-2 RNA (Appendix Figure 3). Thus, although human RNA from saliva degrades without stabilization buffers, SARS-CoV-2 RNA remains protected even at warm temperatures suitable for nuclease activity.

**Figure 2:**
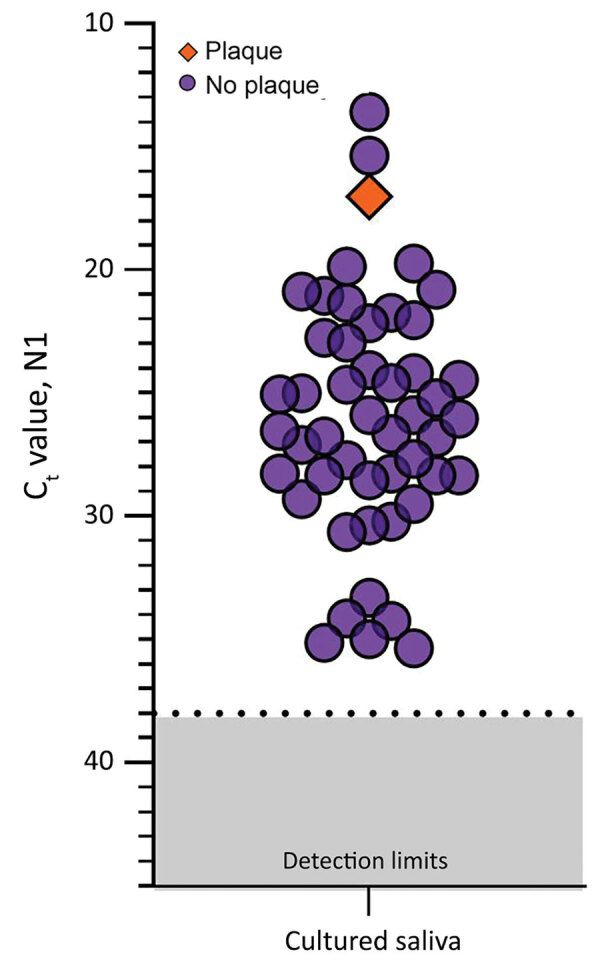
Detection of severe acute respiratory syndrome coronavirus 2 (SARS-CoV-2) in saliva samples tested for infectious SARS-CoV-2. SARS-CoV-2 N1 detection (C_t_ values) measured by quantitative reverse transcription PCR for each saliva sample incubated with Vero-E6 cells for 72 hours. The orange diamond depicts the only sample that produced plaque-forming units (titer increase of 3.79 × 10^4^ PFU/mL; purple circles indicate samples that did not produce plaque-forming units by 72 h after inoculation; dashed lines indicate C_t_ 38 (the cutoff for sample positivity); gray shading indicates C_t_s below the limit of detection. C_t_, cycle threshold.

Because saliva has antiviral properties (*9*,*10*), we explored the infectiousness of SARS-CoV-2 in saliva samples. We inoculated Vero-E6 cells with 49 saliva samples with higher virus RNA titers (C_t_ range 13.57–35.32, median 26.01; Appendix Figure 4) because others have shown that SARS-CoV-2 isolation is uncommon from samples with low virus RNA titers (*11*,*12*; M.D. Folgueira, unpub. data, https://www.medrxiv.org/content/10.1101/2020.06.10.20127837v1). By 72 hours after inoculation, C_t_ values were reduced in 9 (18.7%) of the 49 cultured saliva samples tested by qRT-PCR (−12.90, −11.53, −4.30, −3.68, −3.49, −2.88, −2.81, −2.66, −2.40). Although these findings suggest an increased number of SARS-CoV-2 RNA copies by 72 hours, they may not definitively demonstrate active virus replication. For instance, C_t_ reductions could also result from sampling artifacts or assay variations (disparities in inoculation, RNA extraction, and qRT-PCR). To determine whether this amplification resulted from detectable, active virus replication, we performed plaque assays in triplicate with cellular lysate from 72 hours after inoculation. Only 1 of these 9 samples produced plaque-forming units; titer increased 3.79 × 10^4^ PFU/mL at 1 hour and at 72 hours after inoculation (Figure 2). This finding suggests that increased SARS-CoV-2 genome copies identified by qRT-PCR may fall below the limit of detection in plaque assay sensitivity (100 PFU/mL) until a certain reduction in C_t_ is reached (e.g., C_t_ reduction ≤12.90) or that components of saliva possibly inhibit active viral particle production and release in vitro. A similar result has been observed when attempting to perform plaque assays of virus from the colon (*13*), despite studies showing that SARS-CoV-2 infects gut enterocytes (*14*).

## Conclusions

The cost of commercial tubes specialized for saliva collection and SARS-CoV-2 RNA stabilization (>$7/tube) (Table) can be prohibitive for mass testing. Inexpensive saliva-based testing methods are urgently needed to help reach the capacity required to safely reopen schools and workplaces. We demonstrate the stability of SARS-CoV-2 RNA detection in saliva stored for prolonged periods in a variety of settings, which indicates that saliva can be simply collected without the need for additives.

**Table:** Possible saliva collection devices for severe acute respiratory virus coronavirus 2 RNA testing

Tube type	Collection	Buffer type	Cost per sample, USD	Manufacturer
Oragene•Dx collection device (OGD-510)*	Funnel	Ethanol <24%; Tris 1%–5% (host DNA stabilization)	28.00	Genotek, https://www.dnagenotek.com
Samplify SD-3000	Funnel	Dry preservative; sodium dodecyl sulfate <1%	24.00	Samplify (URL not available)
Saliva collection kit	Funnel	Unknown	22.47	IBI Scientific, https://www.ibisci.com
SDNA-1000 small tubes*	Wide-mouth tube	Ethanol 10%–25%; Tris 1%–5%; thiocyanic acid:guanidine (1:1) 25%–50%; pH 7.9–8.3	17.99	Spectrum Solutions, https://spectrumsolution.com
Saliva RNA Collection and Preservation Device	Wide-mouth tube	Unknown liquid, colorless, odorless	18	Norgen (Biotek), https://norgenbiotek.com
Liquid biopsy/spit devices	Complicated unit (various)	Unknown	9–12 each	Oasis Diagnostics, https://4saliva.com
OMNIgene•ORAL saliva collection device (OM-505)*	Funnel	Sodium dodecyl sulfate 1%–5%; glycine,N,N′-trans- 1,2-cyclohexanediylbis [N-(carboxymethyl)-,hydrate 1%–5%; lithium chloride 0.5%–1.5%	9.50	Genotek
GeneFix Saliva DNA/RNA Collection	Funnel	Unknown liquid, colorless	9	Isohelix, https://isohelix.com
DNA/RNA Shield saliva collection kit*	Wide-mouth tube	Unknown liquid, colorless, pH 5.0–7	7.25	Zymo Research, https://www.zymoresearch.com
Saliva collection system	Small beaker	Unknown	Unavailable	Greiner Bio-One, ttps://www.gbo.com
Pedia•SAL Infant/Toddler Salivary Collection	Soother + collector	None	Unavailable	Oasis Diagnostics
Oral swab	Swab	None	1.76	Salimetrics, https://salimetrics.com
Saliva collection aid + cryovial	Straw + 2 mL collection vial	None	1.36/straw, 0.76/vial	Salimetrics
Urine collection cups	Wide-mouth cup	None	0.47	ThermoFisher, https://www.thermofisher.com
Sterile tube, large volume	Wide-mouth tube	None	0.46 (25 mL), 0.38 (5 mL)	Eppendorf, https://www.eppendorf.com
Sterile tube, small volume	Narrow-mouth tube	None	0.16 (2 mL)	ThermoFisher

*Approved by US Food and Drug Administration Emergency Use Authorization for saliva-based diagnostics.

Previous studies have demonstrated the ease with which saliva can be collected into simple, wide-mouth containers (*3*,*15*) and that buffers marketed for RNA stabilization may be detrimental to SARS-CoV-2 detection (S.B. Griesemer et al., unpub data, https://doi.org/10.1101/2020.06.16.20133041). Although some of these buffers are also marketed for virus inactivation, SARS-CoV-2 is still considered a Biosafety Level 2 hazard, meaning that with or without buffer, any saliva sample should still be handled with care. Without the need for RNA stabilization and given the limited evidence of virus replication in saliva samples, affordable alternatives to making testing accessible throughout the country are simple, sterile, nuclease-free plastic containers.

SARS-CoV-2 stability at room temperature and at 30°C permits more affordable collection and transport strategies without the need for expensive cooling strategies. Absence of the requirement for cold chain handling also makes saliva testing easier in regions with limited resources. Thus, one key for meeting mass testing demands is collection of saliva in simple, sterile, nuclease-fee tubes, negating the high costs associated with specialized collection devices.

Appendix Additional methods and results for study of stability of SARS-CoV-2 RNA in nonsupplemented saliva.
