# Supplementary material for: Stability of SARS-CoV-2 RNA in Nonsupplemented Saliva
Source: Emerg Infect Dis. 2021 Apr;27(4):1146–50. doi: 10.3201/eid2704.204199 (PMC8007305; doi:10.3201/eid2704.204199)
Supplement: Appendix — Additional methods and results for study of stability of SARS-CoV-2 RNA in nonsupplemented saliva. [file 20-4199-Techapp-s1.pdf]

Article DOI: https://doi.org/10.3201/eid2704.204199

Stability of SARS-CoV-2 RNA in Nonsupplemented Saliva

Appendix

Methods

RNA extraction and SARS-CoV-2 detection

Saliva samples were self-collected by COVID-19 inpatients and healthcare workers at the Yale-New Haven Hospital (Yale Human Research Protection Program Institutional Review Boards FWA00002571, Protocol ID. 2000027690) (2), into plain wide-mouth containers without the addition of stabilizing buffers. RNA was extracted from saliva samples (1) and tested by RT-qPCR for SARS-CoV-2 RNA (N1) and human *RNase P* (RP) (3) on day of collection (≤12 hours post sample collection) and at various time points after the storage of the remaining, unsupplemented samples at temperatures of -80°C, -20°C, +4°C, room temperature (measured at an average of ~19ºC), or 30°C.

Cell culture

Vero-E6 cells (ATCC) were cultured in Dulbecco’s Modified Eagle Medium (Gibco) supplemented with 10% heat-inactivated fetal bovine serum (VWR), 1% Penicillin/Streptomycin (Gibco), 100 µg/mL gentamicin (Gibco), and 0.5 µg/mL amphotericin B (Gibco). All cells were incubated at 37°C and 5% CO_2. All cell culture experiments were performed in a biosafety level 3 laboratory at Yale University and approved by the Yale University Biosafety Committee.

Saliva inoculation and virus culture in Vero-E6 cells

Saliva samples were diluted 1:1 in 1X Dulbecco’s PBS (Gibco). Diluted saliva samples were incubated for one hour at 37°C with $2.5x10^5$ Vero-E6 cells in a 24-well plate (Corning). Unbound virus was aspirated and the media were replaced. Infected Vero-E6 cells were frozen at -80°C at 1 and 72 hours post-inoculation. Thawed samples were used for plaque assays and RNA extraction. Prior to RNA extraction (1) and RT-qPCR detection of SARS-CoV-2 RNA (3) the Vero-E6 cells from 1 and 72 hours post-inoculation were thawed at room temperature and further

lysed by diluting 1:3 in MagMax Binding Solution (ThermoFisher). RNA was extracted from the two timepoints and tested in RT-qPCR for SARS-CoV-2 N1. We interpreted a Ct reduction >2 as a difference which could potentially be explained by viral replication during the two timepoints.

Plaque assay

Vero-E6 cells were seeded at $4x10^5$ cells/well in 12-well plates (Corning). The following day, media were removed and replaced with 100 μl of 10-fold serial dilutions of thawed 1 hour or 72 hour post-inoculation saliva samples. Plates were incubated at 37°C for 1 hour with gentle rocking every 15 mins. Unbound inocula was aspirated from each well and overlay media (DMEM, 2% FBS, 0.6% Avicel RC-581 (DuPont)) were added to each well. At 48 hours post-infection, plates were fixed with 5-10% formaldehyde for 30 min then stained with crystal violet solution (0.5% crystal violet in 20% ethanol) for 30 mins. Crystal violet solution was then aspirated and plates were washed in tap water to visualize plaques.

Statistical analyses

We fit a linear regression to the experimental stability data to model the change in Ct values of positive samples following stability conditions using the equation below. Let dct be the change in Ct value from fresh testing following each storage condition and let condition be the categorical storage condition (e.g. freeze/thaw, room temperature, 30°C, etc).

dct ~ condition

Robust confidence intervals were simulated from this model using the mvrnorm, in the R package "MASS", and quantile functions. This regression was also used to model the effect of prolonged storage in stability conditions on RP.

For extended timepoint analyses of N1 we used a linear mixed effects model to predict the change in Ct values of positive samples under each stability condition for greater durations of time using the equation below. Let timepoint be the number of days under stability conditions and let sample be the patient number.

dct ~ timepoint + (1|sample)

Confidence intervals were computed for this model using confint.merMod, in the R package "lme4".

Further statistical analyses were conducted in GraphPad Prism 8.0.0 as described in the text and figure legends.

References

1. Ott I, Vogels C, Grubaugh N, Wyllie A. Saliva Collection and RNA Extraction for SARS-CoV-2 Detection v1 (protocols.io.bg3pjymn) [cited 2021 Jan 7]. http://dx.doi.org/10.17504/protocols.io.bg3pjymn

2. Wyllie AL, Fournier J, Casanovas-Massana A, Campbell M, Tokuyama M, Vijayakumar P, et al. Saliva or nasopharyngeal swab specimens for detection of SARS-CoV-2. N Engl J Med. 2020;383:1283–6. PubMed https://doi.org/10.1056/NEJMc2016359

3. Vogels CBF, Brito AF, Wyllie AL, Fauver JR, Ott IM, Kalinich CC, et al. Analytical sensitivity and efficiency comparisons of SARS-CoV-2 RT-qPCR primer-probe sets. Nat Microbiol. 2020;5:1299–305. PubMed https://doi.org/10.1038/s41564-020-0761-6

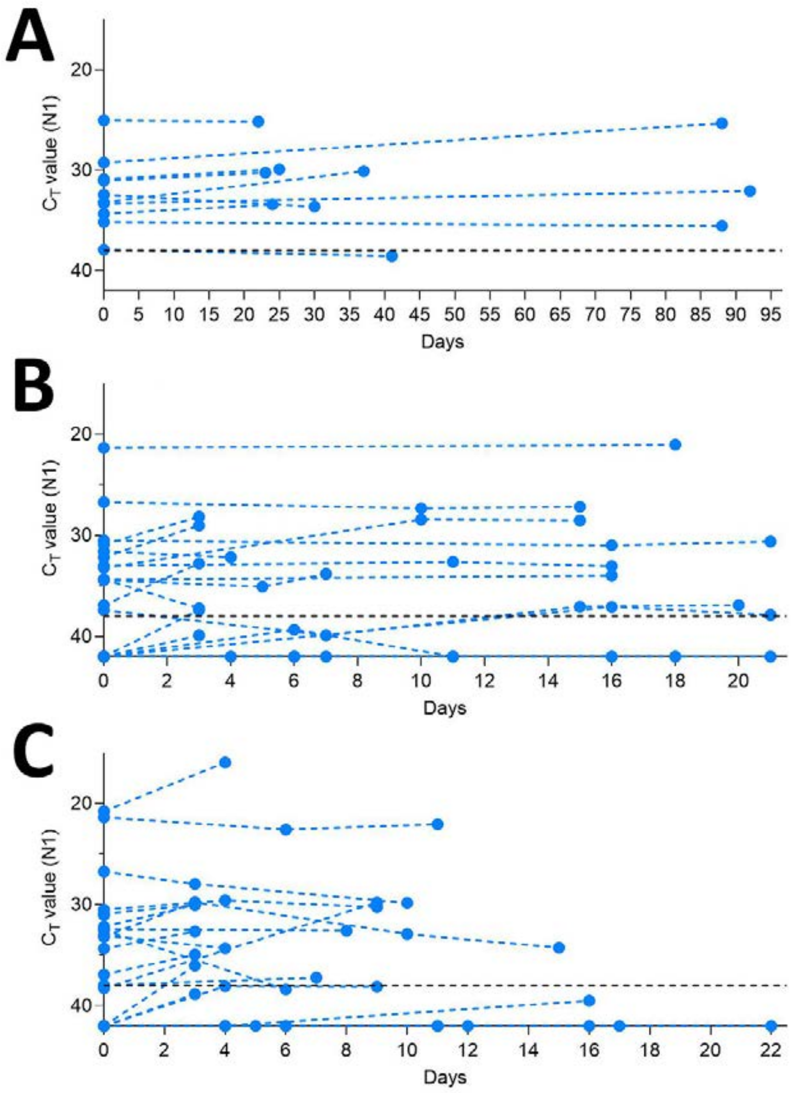

Appendix Figure 1. Stability of SARS-CoV-2 RNA (N1) detection in saliva. SARS-CoV-2 RNA detection in saliva on day of sample collection (0) or after prolonged storage at -80°C, 4°C or 30°C. Ct values from the same original sample are connected by a dotted line. The -80°C and 4°C conditions were found to have a weakly beneficial effect on signal detection by the mixed effects model, while the 30°C condition resulted in a slight increase in Ct. The -80°C storage alone did not cross zero suggesting a mildly stronger effect than the other conditions (95% CI: -0.038, -0.010). The black dashed line represents Ct 38 which we applied as the cut-off to determine sample positivity. Samples that remained not detected (ND) after 45 cycles are depicted as Ct 42.

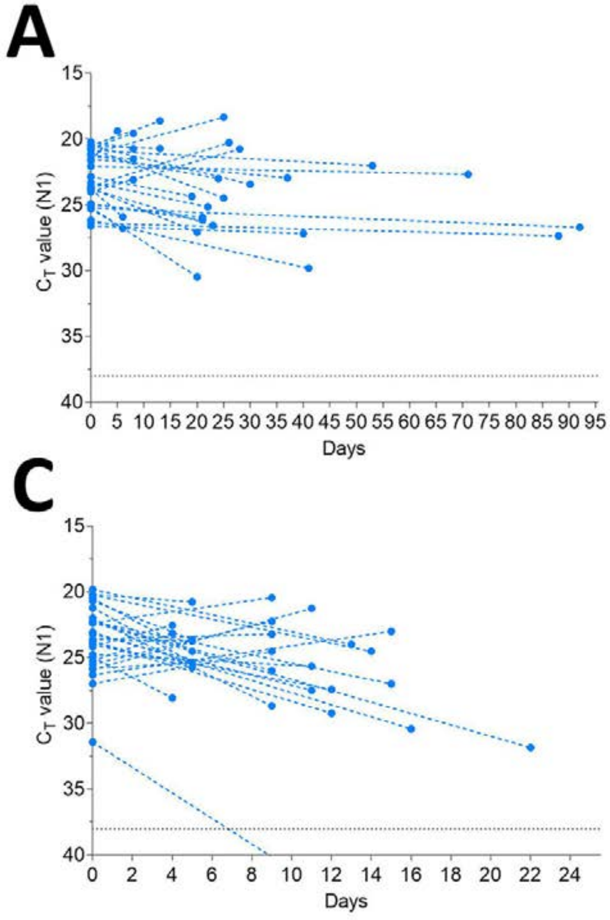

Appendix Figure 2. Detection of human *RNase P* (RP) declines over time when stored in saliva in warmer conditions. Detection of human RP in saliva on day of collection (0) or after prolonged storage at -80°C, 4°C, room temperature (~19°C) or 30°C. Ct values from the same original sample are connected by a dotted line. Prolonged storage at -80°C and 4°C had minimal effect on RP detection with Ct changes of 0.832 (95% CI: -0.402, 2.038) and -0.315 (95% CI: -2.336, 1.687), respectively. However, storage at room temperature (Ct +1.837, 95% CI: 0.468, 3.188) and 30°C (Ct +3.526, 95% CI: 1.750, 5.349) was detrimental to RP, exhibiting a more substantial decrease in signal at these warmer conditions. The black dashed line represents Ct 38 which we applied as the cut-off to determine sample positivity. Samples that remained not detected (ND) after 45 cycles are below the y-axis limit.

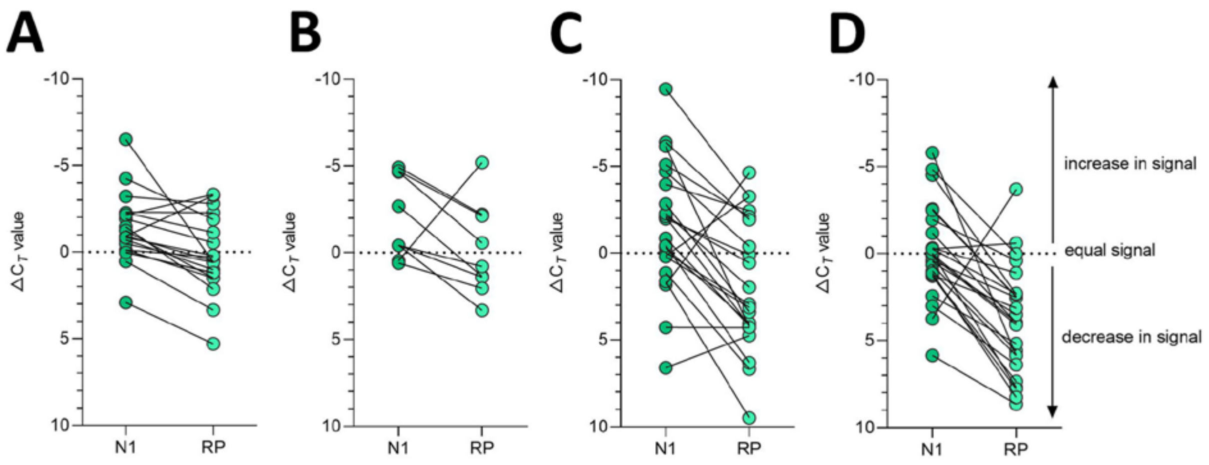

Appendix Figure 3. Detection of SARS-CoV-2 RNA (N1) in saliva remained more stable over time than human *RNase P* (RP). Delta Ct was calculated as the difference in Ct value from the day of saliva collection and after storage at -80°C, 4°C, room temperature (~19°C) or 30°C. Delta Ct values from the same sample are joined by a solid line. While the change in detection of SARS-CoV-2 N1 and RP was similar in saliva samples stored at 4°C (Wilcoxon signed rank test, $p = 0.129$), a greater difference was observed between the change in N1 and RP for samples stored at -80°C ($p = 0.001$), room temperature ($p = 0.001$) and 30°C ($p < 0.0001$).

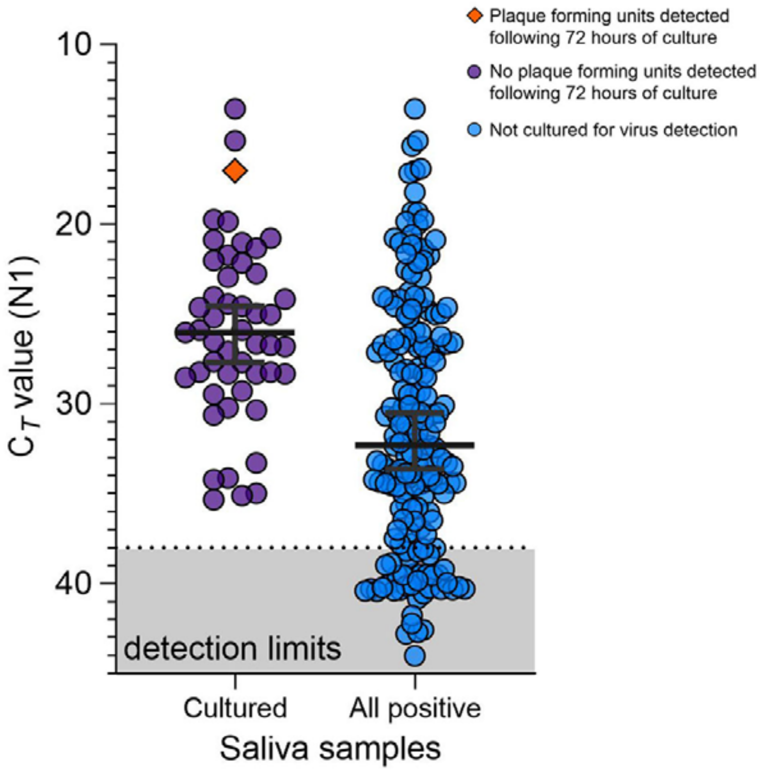

Appendix Figure 4. Saliva samples of relatively high viral load were cultured to evaluate the infectiousness of SARS-CoV-2 in saliva. Saliva samples cultured on Vero-E6 to test for infectious virus were of higher SARS-CoV-2 RNA (N1) load as compared to the overall saliva samples collected by Yale IMPACT (2) which tested positive for SARS-CoV-2 (Mann-Whitney, p = <0.0001). The orange diamond depicts the only sample that produced plaque forming units following 72 hours of culture (PFU; titer increase of 3.79e4 PFU/mL)
